# The Effect of Postoperative Antithrombin Supplementation on the Inflammatory and Coagulation Status of a Surgically Treated Patient With a Malignant Tumor in the Small Pelvis—Case Series

**DOI:** 10.1002/cnr2.70260

**Published:** 2025-06-19

**Authors:** Ranko Lazović, Ognjenka Šarenac

**Affiliations:** ^1^ Clinic for Surgery, Clinical Center of Montenegro University of Montenegro Faculty of Medicine Podgorica Montenegro; ^2^ Clinic for Anesthesia, Resuscitation and Pain Management, Clinical Center of Montenegro University of Montenegro Faculty of Medicine Podgorica Montenegro

**Keywords:** antithrombin, colorectal neoplasms, hemostasis, inflammation, pancreatitis

## Abstract

**Introduction:**

Antithrombin has the function of a natural anticoagulant. It is the main component of the inhibitory anticoagulant system, which, under physiological conditions, opposes the procoagulant mechanisms of hemostasis, preventing the occurrence of a hypercoagulable state, the development of thrombosis, and thromboembolic complications. Antithrombin deficiency, acquired or congenital, leads to the development of a hypercoagulable state and thromboembolic complications. Antithrombin also has a significant anti‐inflammatory, antimicrobial, and antitumor effect. Patients with malignant diseases are often in a hypercoagulable and proinflammatory state due to the nature of the disease itself and the fact that they undergo extensive surgical procedures and chemotherapy as therapeutic modalities. Therapeutic supplementation with antithrombin products could establish normal coagulability and reduce or suppress the inflammatory response. However, previous randomized studies have not shown a long‐term benefit of this approach.

**Case:**

In our female patients who underwent multiple organ resections, with advanced colorectal cancer and acute pancreatitis treated surgically, with a pronounced inflammatory and hypercoagulable response on the first postoperative day, antithrombin supplementation in the first four postoperative days contributed to the establishment of normal coagulability confirmed by the ROTEM analysis while suppressing the inflammatory response followed by inflammatory laboratory parameters.

**Conclusion:**

Compensating for the antithrombin deficit in the first four postoperative days may benefit the specified patient group, with careful monitoring of hemostatic and inflammatory parameters.

## 1 Introduction

During the 80s and 90s, the first studies that examined the inflammatory and coagulation status of patients with malignant diseases, especially in advanced stages of the disease, indicated significant changes. These changes, which included pronounced inflammation due to perturbations of the immune system, hypercoagulability, pronounced fibrinolysis, and an increased risk of thromboembolic complications, highlight the urgent need for further research. These changes are based on different pathophysiological mechanisms: First of all, molecular, humoral, cellular, and vascular, which, at the level of various tissues and organs, all contribute to the manifestations of paraneoplastic syndrome [1].

Systemic inflammation and coagulation are two inseparable processes with significant mutual bidirectional influence [2, 3, 4]. Any change in the humoral and cellular components of the inflammatory response will inevitably have repercussions on the coagulation system (and vice versa) through a positive feedback mechanism [5]. The complex interaction between inflammation and coagulation is well studied and explained in cases of severe infections, sepsis, and polytrauma, while in malignant diseases, it can represent a potential target site for different therapeutic modalities, such as immune checkpoint inhibitors [3]. Also, the influence of external factors that can stimulate, maintain, or increase the inflammatory and coagulation response in patients with malignancies must not be forgotten. Because these patients often undergo extensive, long‐term, mutilating and repetitive surgical interventions as part of the surgical treatment modalities of the underlying disease, which can be clearly and precisely seen from our case presentation, surgical stress and anesthesia further worsen the already existing imbalance of the immune and coagulation system. In addition, such patients receive different hormonal or chemotherapy, have different catheters placed, and so on all of which increase the risk of thromboembolic complications [5].

## 2 Case Reports

The following three case reports are of patients who were treated at the Center for Digestive Surgery, Clinical Center of Montenegro, from January to December 2021.

### 2.1 Case 1

The first case report refers to a 47‐year‐old female patient who was diagnosed with adenocarcinoma of the sigmoid part of the colon and rectum in 2021.

Specifically, in February 2021, the patient suffered a perforation of the colon at the level of the rectosigmoid transition due to an unidentified lesion, which is why she underwent emergency surgery, according to Hartmann (a terminal colostomy was placed) when tissue samples were sent for pathophysiological analysis. The results of the PH analysis showed moderately differentiated adenocarcinoma (PCI = 21), after which cycles of chemotherapy (Cisplatin, Adriablastin, and Mitomycin) were started until August 2021. In December 2021, colostomy reconstruction was performed. The biopsied deposits on the small intestine were sent for PH processing, which showed the existence of adenocarcinoma in the form of peritoneal dissemination.

Further PET and MRI imaging of the abdomen and pelvis revealed two secondary liver metastases (in Segments VII and II), along with multiple peritoneal implants. The patient underwent chemotherapy for 1 year until cytoreductive surgery with HIPEC was indicated.

Following preoperative preparation, the patient, who was in a condition with leukocyte and neutrophil counts within the reference range (4.48 × 10^9^/L and 1.92 × 10^9^/L, respectively), as well as normal levels of total reactive lymphocytes, immature granulocytes, and metabolically reactive neutrophils, underwent extensive surgery under general endotracheal anesthesia (Table 1). The surgery included anterior lower rectal resection with omentectomy, hysterectomy, and metastasectomy of the liver lesions, followed by cytoreduction using the HIPEC method. Propyluracil was administered at a 30% reduced dose, and cytostatics were applied for 1 h. Inflammatory and coagulation parameters were closely monitored before and after surgery.

**TABLE 1:** Influence of postoperatively administered Kybernin (antithrombin) on the patient's inflammatory and coagulation status.

	Preoperatively	First postoperative day	Second postoperative day	Third postoperative day	Fourth postoperative day	Eighth postoperative day
Leukocyte number	4.48 × 109/L	12 × 10^9^/L	10 × 10^9^/L	Normal	2.83 × 10^9^/L	Bone marrow awakening
Neutrophil number	1.92 × 109/L	11 × 10^9^/L	9 × 10^9^/L	Normal	1.51 × 10^9^/L	
CRP	0.5 mg/L	178 mg/L	110 mg/L	58.4 mg/L	28 mg/L	
Fibrinogen	3 g/L	4.2 g/L	3 g/L	Normal	Normal	
Procalcitonin	0.14 ng/mL	1.2 ng/mL	1.5 ng/mL	1.2 ng/mL	1.2 ng/mL	
IL‐6	10 pg/mL	100 pg/mL	10 pg/mL	10 pg/mL	10 pg/mL	
IL‐10	5 pg/mL	50 pg/mL	50 pg/mL	10 pg/mL	10 pg/mL	
PT	Normal	14 s	Normal	Normal	Normal	
aPTT	Normal	45 s	Normal	Normal	Normal	
D‐dimer	7.58 mg/mL	15 mg/mL	150 mg/mL	100 mg/mL	50 mg/mL	
AT	100%	60%	70%	90%	110%	

Abbreviations: aPTT—activated partial thromboplastin time; AT—antithrombin; PT—prothrombin time.

For the first four postoperative days, the patient received Kybernin (Antithrombin) at a dose of 1500 IU per day, with continuous monitoring of inflammatory and hemostatic parameters to assess its effects on coagulation and inflammation. Preoperative blood analysis showed leukocyte and neutrophil counts within the reference range (4.48 × 10^9^/L and 1.92 × 10^9^/L, respectively), as well as normal levels of total reactive lymphocytes, immature granulocytes, and metabolically reactive neutrophils. CRP and fibrinogen levels were also within normal limits (0.5 mg/L and 3 g/L, respectively). ROTEM analysis indicated optimal normocoagulability, with adequate platelet and fibrinogen contributions to clot strength and stability.

Postoperative laboratory results showed a relative increase in neutrophils, leukocytes, lymphocytes, and immature and reactive forms, while fibrinogen remained within the reference range. CRP levels were elevated to approximately 10 mg/L. This favorable response to treatment provides a promising outlook for the patient's recovery.

On the first postoperative day, a pronounced inflammatory response was observed, characterized by leukocytosis with neutrophilia (12.0 × 10^9^/L and 11.0 × 10^9^/L, respectively) and an increase in immature granulocytes and reactive neutrophils. Elevated CRP and fibrinogen levels (178 mg/L and 4.2 g/L, respectively) were noted, along with prolonged prothrombin time (PT), activated partial thromboplastin time (aPTT) (14 s and 45 s, respectively), and D‐dimer (15 mg/mL). Additionally, antithrombin (AT) activity was significantly reduced to 41.6%.

By the second postoperative day, leukocyte and neutrophil counts (10.0 × 10^9^/L and 9.0 × 10^9^/L, respectively), CRP, and fibrinogen levels (110 mg/L and 3 g/L, respectively) had trended downward. Immature and reactive leukocyte forms normalized, along with PT and aPTT values.

On the third postoperative day, leukocyte counts, their subtypes, fibrinogen levels, and hemostasis parameters (PT, aPTT) had all returned to reference values, while CRP and D‐dimer continued to decrease, reaching 58.4 mg/L.

By the fourth postoperative day, the patient developed pancytopenia, including leukopenia and neutropenia (2.83 × 10^9^/L and 1.51 × 10^9^/L, respectively), due to bone marrow aplasia as a side effect of cytostatic therapy. Consequently, Neupogen (filgrastim) was administered. CRP levels remained elevated at approximately 28 mg/L. Around this time, rectal anastomotic dehiscence occurred, leading to a controlled fistula and pelvic sepsis. However, the timely administration of Kybernin (Antithrombin) 4 days after the diagnosis of dehiscence resulted in a significant clinical and biochemical improvement in inflammatory markers, underscoring the effectiveness of the treatment and instilling confidence in the chosen course of action.

Bone marrow recovery commenced on the eighth day following Neupogen (filgrastim) administration.

The patient was subsequently discharged home.

### 2.2 Case 2

Upon admission to the Gastroenterohepatology Department of the Internal Clinic of the Clinical Center of Montenegro, a 65‐year‐old female patient presented with a range of distressing symptoms, including severe stomach pain, bloating, nausea, and vomiting, which had persisted for several days. Despite her discomfort, she was conscious and oriented. However, her condition was concerning, with signs of respiratory insufficiency, hypotension, and tachycardia. The auscultatory finding over the lungs was a weakened breath murmur; the abdomen was above the level of the chest, soft, and painfully sensitive to palpation in the region of the epigastrium and the right rib cage. Diagnostic tests, including a native X‐ray of the abdomen, ultrasound, and MSCT of the abdomen, confirmed the signs of necrotic pancreatitis with ascites and bilateral pleural effusion. The patient was promptly transferred to the Center for Digestive Surgery of the Clinical Center of Montenegro for further evaluation and treatment.

The patient's laboratory findings (Table 2) revealed a severe inflammatory response, with significantly elevated levels of leukocytes and neutrophils (15.98 × 10^9^/L and 12.62 × 10^9^/L, respectively), as well as an increase in immature granulocytes, neutrophils with stronger granulation, and metabolically reactive neutrophils. CRP, fibrinogen, procalcitonin, lipase, and α amylase were all markedly elevated (301 mg/L, 5.9 g/L, 4 ng/mL, 1020 IU/L, and 897 IU/L, respectively), and calcium was decreased (1.52 mmol/L). Hemostasis analyses showed prolonged PT (15 s), prolonged aPTT (43 s), and higher D‐dimer values (10.26 mg/L), indicating a serious and complex medical condition. Under general endotracheal anesthesia, partial pancreatic necrosectomy, cholecystectomy, and omentectomy were performed. During the first four postoperative days, during which Kybernin (Antithrombin) was administered at a dose of 1500 IU per day, inflammatory and hemostatic parameters were monitored, and its influence on inflammation and coagulation status was assessed.

**TABLE 2:** Influence of postoperatively administered Kybernin (antithrombin) on the patient's inflammatory and coagulation status.

	Preoperatively	First postoperative day	Second postoperative day	Third postoperative day	Fourth postoperative day
Leukocyte number	15.98 × 10^9^/L	Normal	Normal	Normal	Normal
Neutrophil number	12.62 × 10^9^/L	Normal	Normal	Normal	Normal
CRP	301 mg/L	248 mg/L	220 mg/L	150 mg/L	Normal
Fibrinogen	5.9 g/L	4.4 g/L	4 g/L	Normal	Normal
Procalcitonin	4 ng/mL	2 ng/mL	1.4 ng/mL	1.1 ng/mL	Normal
IL‐6	1000 pg/mL	1000 pg/mL	500 pg/mL	100 pg/mL	50 pg/mL
IL‐10	2000 pg/mL	1000 pg/mL	300 pg/mL	300 pg/mL	10 pg/mL
PT	15 s	Normal	Normal	Normal	Normal
aPTT	43 s	Normal	Normal	Normal	Normal
D‐dimer	10.26 mg/L	Normal	4 mg/L	Normal	Normal
AT	89%	60%	80%	90%	100%

Abbreviations: aPTT—activated partial thromboplastin time; AT—antithrombin; PT—prothrombin time.

On the first postoperative day, the finding of leukocytes and neutrophils was within the reference range (8.5 × 10^9^/L and 7.0 × 10^9^/L, respectively), with normalized values of immature granulocytes, neutrophils with stronger granulation, and reactive neutrophils, indicating a positive response to the postoperative condition. The CRP, fibrinogen, and procalcitonin values decreased (248 mg/L, i.e., 4.4 g/L). Findings of PT, aPTT, and thrombin time (TT) were within reference values. ROTEM showed optimal normocoagulability with supraphysiological fibrin polymerization (MCF in Fibtem 29 mm).

These results indicate a significant improvement in the patient's condition. Continuing the positive trend, the patient's inflammation and hemostasis parameters remained within the normal range on the second postoperative day. CRP, procalcitonin, fibrinogen, and D‐dimer levels continued to decrease at 220 mg/L, 1.4 ng/L, 4 g/L, and 4 mg/L, respectively.

On the third postoperative day, the values of CRP and procalcitonin continued to decrease (150 mg/L and 1.1 ng/mL, respectively), and hemostasis parameters (PT, aPTT, INR, TT, D‐dimer, and ROTEM) showed optimal normocoagulability.

On the fourth postoperative day, inflammation parameters were normalized, and coagulation parameters were normalized in the ROTEM test.

Additionally, preoperative levels of IL‐6 and IL‐10 were markedly elevated and began to decline on the second postoperative day, suggesting a role of AT in modulating the inflammatory response.

As a result of these positive developments, the patient was discharged home, marking a successful conclusion to their postoperative care.

### 2.3 Case 3

The patient, a 36‐year‐old with ovarian malignancy, has been under our care since 2021 when she was diagnosed with ovarian epithelial cancer—Stage 2/3. Following pathophysiological verification, the patient underwent a meticulously performed surgery in February 2021; a total hysterectomy with bilateral adnexectomy and partial omentectomy was carried out. Six cycles of chemotherapy were administered postoperatively. The patient's condition was regularly monitored by an oncologist, gynecologist, and radiologist. Nine months after the primary treatment, a CT scan revealed a tumoral change in the small pelvis with infiltration of the sigmoid colon, proximal rectum, and posterior fornix of the vagina with retroperitoneal lymphadenopathy of the preaortic, paracaval, and obturator groups of lymph nodes. Due to the suspected peritoneal dissemination of cancer, the patient underwent a second surgical resection of the rectum and colon, with omentectomy and lymphadenectomy of the aforementioned lymph nodes. In the same act, an extempore biopsy of the parietal peritoneum was performed; the findings were benign. Given the complexity and extent of the surgical procedure performed and the very nature of the underlying disease, parameters of the patient's inflammatory and coagulation status were monitored preoperatively and in the first 5 days after the surgical procedure, with special reference to the first 4 days postoperatively when the Kybernin (Antithrombin) was administered.

In this case, we highlight the crucial role of postoperatively administered Kybernin (Antithrombin) in managing the patient's inflammatory and coagulation status, underscoring the importance of our collective efforts in the patient's recovery.

We closely tracked the changes in the patient's inflammatory markers (leukocytes, neutrophils, immature granulocytes, CRP, fibrinogen, procalcitonin) and hemostasis parameters (antithrombin levels, D‐dimer, aPTT, PT, and all the parameters of the ROTEM method).

Immediately before the operation, mild leukocytosis and neutrophilia were recorded (12.55 × 10^9^/L and 10.75 × 10^9^/L, respectively), with elevated values of CRP, fibrinogen, D‐dimer, PT, and aPTT (69.8 mg/L, 6.3 g/L, 1.3 mg/L, 13.8 s, and 39.7 s respectively). Two hours after the operation was completed, the mentioned parameters were checked, and there was a marked increase in leukocytes, neutrophils, and CRP (Le 42.0 × 10^9^/L, 40.0 × 10^9^/L and 99.8 mg/L, respectively). Hemostasis analyses, primarily ROTEM parameters, showed hypercoagulability (PT was 8 s, ROTEM in Fibtem extended A5 29 mm, A10 33 mm, A20 38 mm, and MCF 40 mm).

On the first postoperative day, Kybernin (Antithrombin) was administered 1500 IU daily for 3 days. The level of antithrombin was monitored daily on the first, second, and third postoperative days (69.1%, 87.2%, and 104.7%, respectively). The normalization of inflammatory parameters was observed as early as the third postoperative day and from the third day of Kybernin (Antithrombin) administration. Leukocytes, neutrophils, and fibrinogen were within the reference values, and CRP was reduced to 58.2 mg/L. The patient's positive response to Kybernin (Antithrombin) is a reassuring indication of its efficacy in stabilizing inflammatory and hemostatic status.

On the fourth postoperative day, normalization of inflammatory parameters and ROTEM analysis was demonstrated.

On the fifth postoperative day, as part of the blood count, leukocytes, neutrophils, immature granulocytes, and fibrinogen were within the reference range. At the same time, CRP continued with a downward trend (26.7 mg/L, then 18.4 mg/L) (Table 3). A repeated ROTEM test after three doses of Kybernin (Antithrombin) showed optimal normocoagulability with supraphysiological fibrin polymerization.

**TABLE 3:** Influence of postoperatively administered Kybernin (Antithrombin) on the patient's inflammatory and coagulation status.

	Preoperatively	Third postoperative day	Fifth postoperative day
Leukocyte number	12.55 × 10^9^/L	Normal	Normal
Neutrophil number	10.75 × 10^9^/L	Normal	Normal
CRP	69.8 mg/L	58.2 mg/L	18.4 mg/L
Fibrinogen	6.3 g/L	Normal	Normal
Procalcitonin	4.5 ng/mL	0.5 ng/mL	0.05 ng/mL
IL − 6	1000 pg/mL	50 pg/mL	10 pg/mL
IL—10	2000 pg/mL	100 pg/mL	10 pg/mL
PT	13.8 s	Normal	Normal
aPTT	39.7 s	Normal	Normal
D‐dimer	1.3 mg/L	Normal	Normal
AT	60%	90%	110%

Abbreviations: aPTT—activated partial thromboplastin time; AT—antithrombin; PT—prothrombin time.

With the patient's condition significantly improved, the decision was made to discharge her home.

## 3 Discussion

The imbalance between procoagulant and anticoagulant mechanisms causes disruption of the coagulation system, which results in hypercoagulability. At the same time, the inflammatory response worsens the imbalance by favoring the activation of coagulation factors [6, 7]. The interaction between tissue factor and coagulation factors leads to enhanced thrombin generation, which further exerts a whole series of procoagulant effects [8].

Beyond its role in fibrinogen breakdown, thrombin is a key regulator of immune responses. Through protease‐activated receptors (PAR), it influences immune cells, fibroblasts, platelets, and endothelial cells, stimulating growth factor secretion and cell migration [1]. This multifaceted function of thrombin, stimulating growth factor secretion and cell migration, is indeed intriguing. Endothelial activation promotes leukocyte recruitment, particularly neutrophils and monocytes, facilitated by von Willebrand factor, leading to enhanced granulocyte secretion and inflammatory responses involving T lymphocytes and neutrophils [9, 10].

Postoperative coagulation, a critical aspect of patient care, is significantly influenced by thrombin. Surgical trauma in cancer patients triggers a strong proinflammatory and procoagulant response. Inflammatory cells release cytokines that stimulate endothelial secretion of mediators and growth factors, increasing platelet aggregation and thrombin generation. Postoperatively, fibrinogen levels decrease due to hemodilution, while impaired fibrinolysis results from elevated plasminogen activator inhibitor‐1 (PAI‐1), driven by TNFα, IL‐1, and IL‐6 [11, 12, 13]. This procoagulant state is exacerbated by reduced plasminogen activity and synthesis [13, 14]. Studies by Khafagy et al. and Nguyen confirmed significant postoperative reductions in antithrombin, protein C, and protein S levels [12, 14]. Administration of antithrombin, a member of the serpin family, either as recombinant or plasmatic form in such patients could suppress the pronounced inflammatory response and establish a disturbed balance between procoagulant and anticoagulant mechanisms, reducing procoagulant and stimulating anticoagulant mechanisms [15, 16].

Antithrombin is a glycoprotein with a fascinating structure. Its 432 amino acids are meticulously arranged into three beta and nine alpha envelopes, housing a central reactive loop. This intricate design, synthesized in the liver without needing vitamin K, produces a plasma concentration of 100–150 μg/mL, that is, 2–3 μmol/L [17, 18, 19]. AT, along with proteins C and S, is essential in coagulation, primarily inhibiting serine proteases through heparin‐AT interactions and modulating proteins C and S [20]. It inhibits thrombin and factor X via its reactive domain, accounting for 80% of its activity, and also affects factors IXa, VIIa, XIa, XIIa, plasmin, trypsin, and kallikrein [17, 18]. Heparin enhances AT's anticoagulant effect by up to 1000 times by increasing its binding affinity to thrombin [21, 22, 23].

AT also exerts anti‐inflammatory effects through interactions with proteoglycans and inhibitory proinflammatory receptors [24]. It binds to endothelial and leukocyte proteoglycans like heparan sulfate and syndecan four while inhibiting tissue factor, TNFα, and cytokine expression via CD13, CD300f, and LRP‐1 receptors [25]. Additionally, AT suppresses NF‐κB‐controlled genes, thereby regulating immune responses and cellular differentiation [26, 27].

AT exhibits antimicrobial activity against bacteria, viruses, fungi, and parasites. It blocks bacterial adhesion to heparin‐binding structures and inhibits viral replication via NF‐κB, heat shock proteins, BMP2, and JUN [28, 29]. The human antithrombin isoform, hβAT, is crucial in reducing inflammatory responses and protecting against organ damage during bacterial infections. It achieves this by inhibiting NF‐κB activation and modulating immune signaling pathways. Additionally, Papareddy et al. revealed potential antimicrobial therapeutic properties of antithrombin, showing that in a lethal *Escherichia coli* infection model, hβAT significantly improved survival rates and reduced bacterial burden [28].

Antithrombin deficiency, a significant risk factor for venous thromboembolism, carries an estimated annual thrombosis risk of up to 2.3%, making it one of the most severe forms of inherited thrombophilia. Even minor reductions in antithrombin levels can escalate thrombotic risk, underscoring the necessity of accurate diagnosis and management strategies in the face of this grave condition [29]. Pharmaceutical AT is plasma‐derived (Thrombate III, Kybernin) and recombinant (ATryn). Plasma AT is used for thromboembolism prophylaxis and treatment in AT‐deficient patients, including perioperative and pregnancy‐related cases. Recombinant AT is approved only for perioperative or peripartum prevention in congenital AT deficiency [30].

AT is used beyond approved indications in various conditions due to its anticoagulant, anti‐inflammatory, antimicrobial, and anticancer properties, though results vary across studies [30, 31]. Takashi et al. reported a significant drop in AT levels and platelet counts post‐gynecological surgery, lasting up to 7 days [32], but there is no consensus on AT use in acquired deficiency, necessitating further research.

In our case reports, three female patients—two with malignancies and one with acute pancreatitis—underwent extensive organ resections and chemotherapy. Preoperative labs showed mild leukocytosis, neutrophilia, elevated CRP and fibrinogen, normal procalcitonin and AT, slightly increased coagulation markers, and elevated D‐dimer. These findings suggest a preexisting state of inflammation and a potential risk of thrombosis. The patients' conditions and treatments provide a relevant context for understanding the effects of AT treatment. Postoperatively, the patients showed a significant improvement in their condition. AT treatment, administered for 4 days based on a calculated dose, successfully stabilized inflammatory markers, normalized leukocytes, neutrophils, fibrinogen, and coagulation parameters, and reduced CRP by nearly 50%. ROTEM confirmed normocoagulability, with AT levels rising above 100%, providing a reassuring sign of successful treatment. In the further course, from the 3rd to the 5th postoperative day, the trend of regulating hemostatic balance continued with a further decrease in inflammatory parameters and a controlled inflammatory response.

Although directly comparable studies are limited, our findings align with existing literature, highlighting the critical role of AT supplementation in mitigating postoperative coagulopathy and improving clinical outcomes. Supporting our observations, Kuroda et al. demonstrated that patients receiving AT supplementation had significantly lower rates of posthepatectomy liver failure compared to those who did not (16.3% vs. 44.2%, *p* < 0.01), suggesting that AT administration may effectively reduce postoperative coagulopathy [33]. Similarly, a meta‐analysis of available data showed that AT supplementation in adults with acute lymphoblastic leukemia was associated with a significantly lower risk of venous thromboembolism compared to patients who did not receive thromboprophylaxis [34].

Beyond hepatic surgery, low postoperative AT levels have been linked to adverse outcomes, particularly in elderly patients, further justifying its targeted supplementation in select populations [35]. Preoperative AT administration has been shown to effectively prevent heparin resistance and maintain higher postoperative AT levels [36]. However, the role of AT supplementation in cardiac surgery remains a topic of debate. While AT is commonly administered to counteract heparin resistance during cardiopulmonary bypass (CPB), its broader efficacy is uncertain. Some studies suggest that AT supplementation reduces thrombin generation and fibrinolysis without significantly affecting platelet activation or inflammatory responses [36]. Furthermore, fluctuations in AT activity within the first 24 h postoperatively have been identified as predictors of intensive care unit (ICU) mortality in critically ill patients. In support of its potential benefits, Gando et al. reported that AT supplementation improved coagulation and inflammatory markers, which may contribute to better long‐term survival [37].

Randomized clinical studies (Blauhut, Vinazzer) confirmed AT benefits in stabilizing coagulation in septic DIC patients, whereas heparin alone or combined with AT showed no such effects [38, 39]. The KyberCept study, while initially finding no mortality difference between the placebo and AT‐treated groups after heparin administration, ultimately revealed a promising outcome. Further analysis showed that AT treatment, when used alone, led to a significant reduction in 28‐day mortality, offering a glimmer of hope in the fight against coagulation‐related mortality [40].

## 4 Conclusion

Supplementation with AT preparations in specific subpopulations of patients with hypercoagulable and hyperinflammatory status and acquired AT deficiency significantly reduces or even suppresses the inflammatory response while simultaneously establishing normocoagulability defined by the ROTEM method. Based on our findings, administering AT in the first four postoperative days may benefit selected subgroups of patients in doses sufficient to increase AT activity above 100%. However, further studies with larger sample sizes and appropriate controls are needed before this approach can be considered a standard treatment.

## Author Contributions

Conceptualization: Ranko Lazovic and Ognjenka Sarenac. Writing – original draft preparation: Ognjenka Sarenac. writing – review and editing: Ranko Lazovic. Supervision: Ranko Lazovic. All authors have read and agreed to the published version of the manuscript.

## Ethics Statement

The patients gave consent for the use of de‐identified information for public scientific dissemination.

## Conflicts of Interest

The authors declare no conflicts of interest.

## Data Availability

The data that support the findings of this study are available on request from the corresponding author. The data are not publicly available due to privacy or ethical restrictions.
